# Effects of a *Fusarium* Toxin-Contaminated Maize Treated with Sodium Sulfite on Male Piglets in the Presence of an LPS-Induced Acute Inflammation

**DOI:** 10.3390/toxins10100419

**Published:** 2018-10-18

**Authors:** Anh-Tuan Tran, Jeannette Kluess, Andreas Berk, Marleen Paulick, Jana Frahm, Dian Schatzmayr, Susanne Kersten, Sven Dänicke

**Affiliations:** 1 Institute of Animal Nutrition, Friedrich-Loeffler-Institut (FLI), Federal Research Institute for Animal Health, Bundesallee 37, 38116 Braunschweig, Germany; trantuan05@gmail.com (A-T.T.); andreas.berk@fli.de (A.B.); marleen.paulick@arcor.de (M.P.); jana.frahm@fli.de (J.F.); susanne.kersten@fli.de (S.K.); sven.daenicke@fli.de (S.D.); 2 BIOMIN Holding GmbH, BIOMIN Research Center, Technopark 1, 3430 Tulln, Austria; dian.schatzmayr@biomin.net

**Keywords:** deoxynivalenol, sodium sulfite, detoxification, piglets, lipopolysaccharide

## Abstract

We investigated the effects of feeding sodium sulfite (SoS) treated uncontaminated and *Fusarium* contaminated maize in a porcine lipopolysaccharide (LPS) challenge model. Eighty piglets (7.59 ± 0.92 kg body weight [BW]) were equally assigned to one of four experimental diets containing 10% maize, either uncontaminated and untreated (CON−, 0.09 mg deoxynivalenol [DON]/kg diet) or uncontaminated and SoS-treated (CON+, wet-preserved with 5 g SoS/kg maize; 0.05 mg DON/kg diet), or prepared with 10% of a *Fusarium* contaminated maize containing mainly deoxynivalenol (DON), either contaminated and untreated (FUS−, 5.36 mg DON/kg diet), or contaminated and SoS-treated (FUS+, wet-preserved with 5 g SoS/kg maize; 0.83 mg DON/kg diet). At day 42 of experiment, ten pigs of each group were injected intraperitoneally with either 7.5 µg LPS/kg BW or placebo (0.9% NaCl). At 120 min after injection, blood samples were collected to analyse TNF-α, hematological profile, clinical biochemistry as well as the redox status. A significant increase in body temperature and cytokine TNF-α concentration was observed in the LPS-injected piglets. Results for hematology, clinical chemistry and redox status indicate no effects of SoS treatment, with exception of neutrophil counts being significantly more pronounced after feeding the SoS treated FUS maize. In conclusion, SoS treatment of maize did not modulate the LPS-induced acute inflammation.

## 1. Introduction

The mycotoxin deoxynivalenol (DON) is mainly produced by *Fusarium* species and commonly found in agricultural commodities and finished feed [1]. Pigs are the most sensitive species with respect to DON, with prominent reduction of feed intake resulting in reduced weight gain [1]. As *Fusarium* infection of cereal strongly depends on the climate conditions, especially on temperature and humidity, such mycotoxin contamination cannot be completely avoided. Moreover, DON is rather stable under milling, processing and heating [2]. Therefore, decontamination strategies are needed in order to detoxify DON contaminated cereal grains before mixing into the diet [3]. Although many methods of decontamination are applied, an effective method at farm level is still lacking. Previous studies suggested that wet-preservation of *Fusarium* toxin-contaminated maize with sodium sulfite (Na_2_SO_3_, SoS) clearly reduced its DON content due to the transformation of DON into DON-sulfonates (DONS), which are considerably less toxic than DON [4,5,6]. Moreover, when piglets consumed SoS-treated maize, a decreased DON- and an increased DONS level was observed in plasma [7]. This was accompanied by an improved feed intake and an increased weight gain of animals [8]. Additionally, the reduced-DON concentrations in plasma were paralleled by lower DON levels in various specimens (urine, bile and liquor) supporting an efficient detoxification with SoS [9]. However, the health-related parameters did not consistently reflect the positive effects of SoS treatment [9].

Similar to SoS, sodium bisulfite (SBS) has been already investigated in various studies and was shown as an effective substance to detoxify DON contaminated cereal grains [6,10,11,12]. On the other hand, SBS also increased the plasma protein concentration, stimulated the functional liver capacity as determined by the ^13^C-methacetin breath test and stimulation ability of peripheral blood mononuclear cells in pigs fed an SBS treated diet indicating side effects of SBS treatment [12,13].

Based on these non-specific effects in general, and the critical role of the liver as secondary immunological organ and its involvement in the initiation and mediation of the acute phase response as an innate immune mechanism in particular, the consequences for inflammatory conditions remain to be elucidated. Experimentally, a strong inflammatory response can be induced by systemic application of lipopolysaccharides (LPS). LPS, acting as an endotoxin, is a major component of the outer membrane of Gram-negative bacteria [14]. LPS is known as a pathogen-associated molecular pattern (PAMP) capable of inducing an acute phase response [15]. Additionally, it has been suggested that LPS possibly interacted with DON and resulted in an upregulation of pro-inflammatory cytokines such as tumor necrosis factor-α (TNF-α) in mice [16,17] as well as in porcine hepatic Kupffer cell cultures [18]. Furthermore, co-exposure to LPS and DON amplified the toxicity of DON in mice through an increase in apoptosis in lymphoid organs (thymus, spleen and Peyer’s patches) when mice were intraperitoneally injected with LPS (0.5 mg LPS/kg body weight [BW]) and DON (25 mg DON/kg BW) [19]. In swine, higher DON content was observed in blood of pigs infused with DON (0.1 mg DON/kg BW) and LPS (7.5 µg LPS/kg BW) [20] as well as an augmentation of lactic acidosis was reported in pigs fed a DON diet (4.59 mg DON/kg feed) infused with LPS (7.5 µg LPS/kg BW) [21].

Based on the reported non-specific effects of SBS on metabolism such effects might also occur as a consequence of a SoS treatment. Moreover, the course of a systemic inflammation might be modified by SoS treatment and further modulated by the discussed interactions between DON and LPS. Therefore, the aim of present study was to investigate the effects of feeding SoS treated uncontaminated and contaminated maize under inflammatory stress conditions.

## 2. Results

### 2.1. Clinical Signs

**Figure 1:**
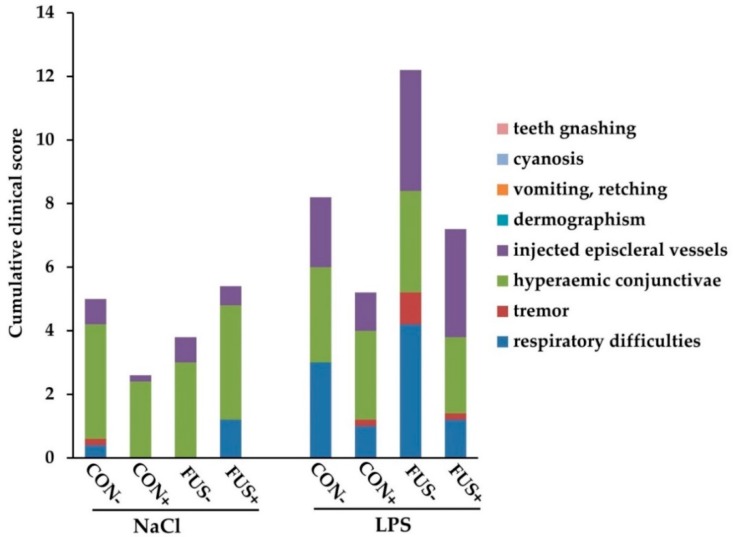
Cumulative clinical score of male castrated piglets fed diets containing untreated control (CON−) or *Fusarium* toxin-contaminated maize (FUS−), or sodium sulfite (SoS) wet-preserved treated control (CON+) and FUS maize (FUS+). Pigs were injected intraperitoneally with either 7.5 µg LPS/kg BW or 0.9% NaCl (LSMeans, *n* = 5). The entire clinical symptoms are presented as the cumulative clinical score (CCS). Each CCS bar consists of maximum 8 symptoms scored at four time points over the entire observation period of 120 min for each group. ANOVA, *p*-values: *p*_maize_ = **0.001**; *p*_treatment_ = **0.034**; *p*_injection_ < **0.001**; *p*_time_ = **0.011**; *p*_maize×treatment_ = 0.940; *p*_maize×injection_ = 0.066; *p*_treatment×injection_ = 0.090; *p*_maize×treatment×injection_ = **0.017**; *p*_maize×treatment×injection×time_ = 0.245.

Typical clinical signs of an inflammatory response were recorded in a time dependent manner and scored for their presence in all experimental groups. The sum of all symptoms was calculated over time and deemed as cumulative clinical score (CCS), whereby the highest achievable score of 88 represents the most severe clinical presentation. Data on CCS are presented in Figure 1. The highest CCS was only 12.2 for the group FUS−/LPS. In contrast, the lowest CCS with 2.6 was found in group CON+/NaCl, whereas the other groups (CON−/NaCl, FUS−/NaCl, FUS+/NaCl, CON−/LPS, CON+/LPS and FUS+/LPS) showed similar values of CCS, which resulted in a significant interaction between maize batch, SoS treatment and LPS (*p*_maize×treatment×injection_ = 0.017). In general, the respiratory rate ranged above the physiological value 30 breaths/min [22] and statistically only a significant time effect (*p*_time_ < 0.001) was apparent. Starting from an initial value of 54.4 ± 2.8 breaths/min at −30 min (LSMeans), the respiratory rate was significantly increased at 15 min (66.0 ± 2.7 breaths/min) and 45 min (64.3 ± 2.7 breaths/min), but returned to its initial rate at 75 min (56.3 ± 2.7 breaths/min) and remained there (105 min: 58.2 breaths/min).

**Figure 2:**
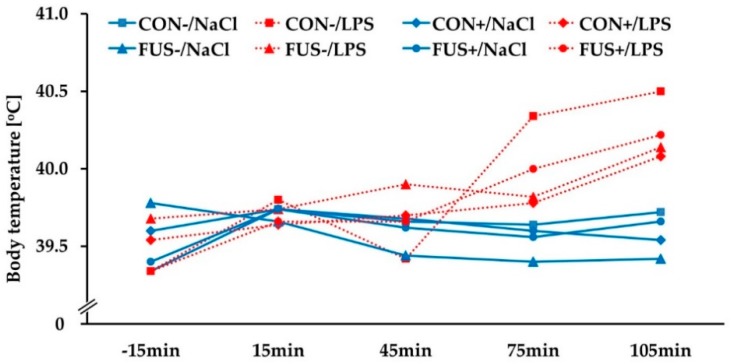
Rectal temperature of male castrated piglets fed diets containing untreated control (CON−, ■) or *Fusarium* toxin-contaminated maize (FUS−, ▲), or sodium sulfite (SoS) wet-preserved treated control (CON+, ♦) and FUS maize (FUS+, ●). Pigs were i.p. injected with either 7.5 µg LPS/kg BW (red dotted lines) or 0.9% NaCl (blue solid lines) (LSMeans, *n* = 5). ANOVA, *p*-values: *p*_maize_ = 0.764; *p*_treatment_ = 0.699; *p*_injection_ = **0.025**; *p*_time_ < **0.001**; *p*_maize×treatment_ = 0.797; *p*_maize×injection_ = 0.764; *p*_treatment×injection_ = 0.467; *p*_maize×treatment×injection_ = 0.965; *p*_maize×treatment×injection×time_ < **0.001**.

As shown in Figure 2, the body temperature was increased in a time-dependent manner due to LPS administration, irrespective of maize batch and SoS treatment. At 105 min after LPS administration, all LPS-injected pigs showed an increase in rectal temperature (*p*_maize×treatment×injection×time_ < 0.001) compared to saline-injected groups.

### 2.2. TNF-α

**Figure 3:**
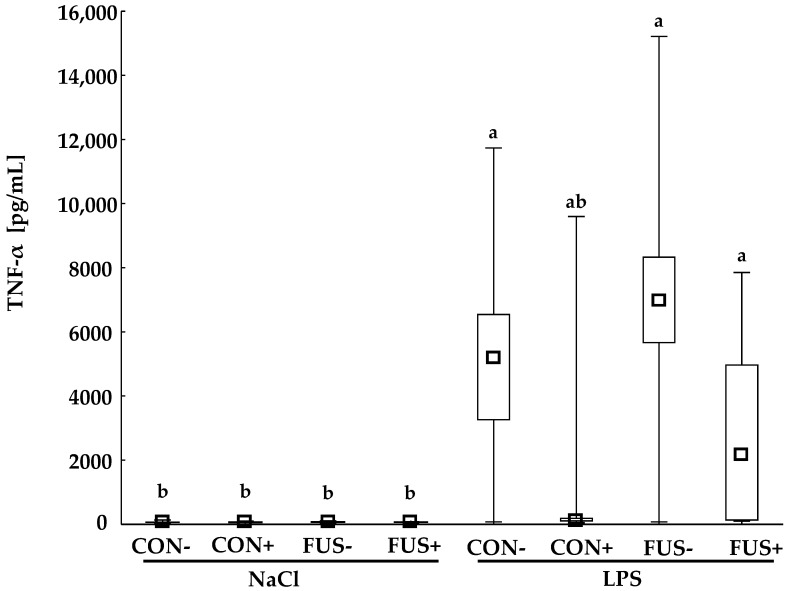
Pro-inflammatory cytokine TNF-α concentration (pg/mL) in serum of male castrated piglets fed diets containing untreated control (CON−) or *Fusarium* toxin-contaminated maize (FUS−), or sodium sulfite (SoS) wet-preserved treated control (CON+) and FUS maize (FUS+). Pigs were i.p. injected with either 7.5 µg LPS/kg BW or 0.9% NaCl. Squares, boxes and whiskers represent medians, 25–75th percentile, and minimum and maximum values, respectively. ^a,b^ Values with no common superscripts are significantly different (Mann-Whitney *U*-test, *p* < 0.05).

Generally, LPS-injected piglets showed significantly higher TNF-α concentrations compared to the saline-injected animals with exception of group CON+/LPS (Figure 3). 

### 2.3. Organ Weights

The relative organ weights relate the absolute organ weights to the live weight of animals (Table 1). Live weight of the pigs fed diets containing untreated *Fusarium* contaminated maize (FUS−) was reduced, compared to the other three groups (CON−, CON+ and FUS+) which exhibited comparable body weights (LSMeans, CON−: 30.1 ± 1 kg; CON+: 30.3 ± 1 kg; FUS−: 26.1 ± 1 kg and FUS+: 30.9 ± 1 kg), resulting in a significant interaction between maize batch and SoS treatment. LPS-injected pigs showed a significantly higher relative spleen weight compared to the saline-injected pigs (pooled LSMeans of LPS-injected pigs: 1.8 ± 0.06 g/kg BW and saline-injected pigs: 1.6 ± 0.06 g/kg BW). Similarly, a significant increase in relative emptied stomach weight was observed in the LPS-injected groups (pooled LSMeans of LPS-injected pigs: 8.6 ± 0.2 g/kg BW and saline-injected pigs: 8.0 ± 0.2 g/kg BW). In addition, the relative emptied stomach weight of feeding group FUS− was increased compared to group FUS+ which appeared similar to the respective weight of control groups (LSMeans, CON−: 7.6 ± 0.3 g/kg BW; CON+: 7.8 ± 0.3 g/kg BW; FUS−: 9.8 ± 0.3 g/kg BW and FUS+: 7.9 ± 0.3 g/kg BW). These relationships resulted in a significant interaction between maize batch and SoS treatment. Furthermore, a significant interaction between maize batch and SoS treatment was also observed for the relative lung weight (*p*_maize×treatment_ = 0.009), whereby FUS+ showed a decrease compared to FUS−, which in turn was comparable to CON+ (LSmeans in g/kg BW, CON−: 12.1 ± 0.8; CON+: 13.8 ± 0.8; FUS−: 14.2 ± 0.8, FUS+: 11.7 ± 0.8).

### 2.4. Haematology

Overall, the red haemogram remained unaffected by either treatment with exception of haemoglobin concentration (Supplementary Table S1). Although LPS administration significantly increased the haemoglobin concentration as compared to its placebo counterparts from 12.1 g/dL to 12.9 g/dL, haemoglobin was still in the physiological range (10.8–14.8 g/dL).

Results for the white differential blood count are presented in Table 2. The total leukocyte counts of LPS-challenged pigs were strongly decreased in some groups (CON−/LPS, FUS−/LPS) even below the physiological reference range (10–22 × 10^9^/L). However, total leukocytes of CON+/LPS were seemingly not affected, which resulted in a significant interaction between maize batch, SoS treatment and LPS injection. Similar significant relationships were also observed for lymphocytes (*p*_maize×treatment×injection_ = 0.012) and monocytes (*p*_maize×treatment×injection_ = 0.011). A further interaction between maize batch and SoS treatment was found for the segmented neutrophils due to an increase in the feeding group FUS+ when compared to other feeding groups (LSMeans, CON−: 3.5 ± 0.5 × 10^9^/L; CON+: 3.1 ± 0.5 × 10^9^/L; FUS−: 3.1 ± 0.5 × 10^9^/L and FUS+: 5.2 ± 0.5 × 10^9^/L). Similarly, the feeding group FUS+ showed a significantly higher concentration of banded neutrophils with 0.8 ± 0.1 × 10^9^/L, while the other feeding groups (CON−, CON+ and FUS−) remained unaltered with 0.5 ± 0.1 × 10^9^/L, 0.5 ± 0.1 × 10^9^/L and 0.4 ± 0.1 × 10^9^/L, respectively. Moreover, both neutrophils (segmented and banded neutrophils) were decreased in all LPS-injected piglets, while the eosinophils remained unaffected by either treatment.

### 2.5. Clinical Chemistry

Total protein and albumin remained unaffected by any treatment (Table 3). Activities of glutamate dehydrogenase (GLDH) were below the LOD (1.0 U/L) in all groups (data not shown). The activity of aspartate aminotransferase (AST) was recorded to be above the reference range of 35 U/L [21] in all groups. The AST activity increased in LPS-injected pigs fed CON diets (CON/LPS) compared to the respective saline-injected pigs (CON/NaCl), whereas the opposite was detected in the FUS fed pigs (LSMeans, CON/NaCl: 38.3 ± 2.9 U/L; CON/LPS: 48.0 ± 2.9 U/L; FUS/NaCl: 49.0 ± 2.9 U/L and FUS/LPS: 45.5 ± 2.9 U/L) as indicated by the significant interaction between maize batch and LPS. In addition, in LPS-injected pigs, significantly increased γ-glutamyltransferase (γ-GT) activity (pooled LSMeans of NaCl injection: 25 U/L and LPS injection: 27.8 U/L) and total bilirubin concentrations (pooled LSMeans of NaCl injection: 2.8 µmol/L and LPS injection: 3.5 µmol/L) were determined.

### 2.6. Redox Status

**Figure 4:**
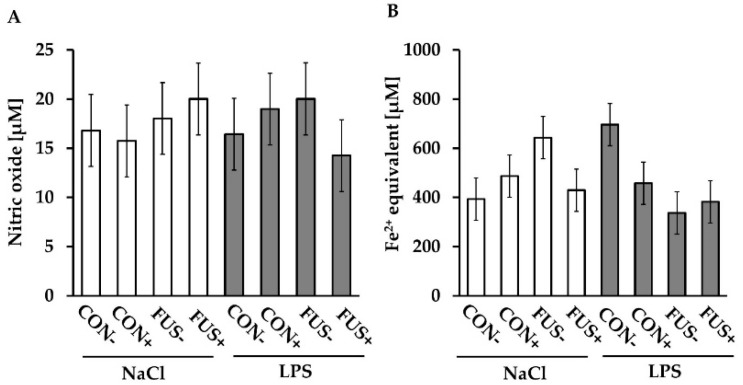
Nitric oxide (NO) production (**A**) and ferric reducing ability (FRA) of serum (**B**) of male castrated piglets fed diets containing untreated control (CON−) or *Fusarium* toxin-contaminated maize (FUS−), or sodium sulfite (SoS) wet-preserved treated control (CON+) and FUS maize (FUS+). Pigs were i.p. injected with either 7.5 µg LPS/kg BW or 0.9% NaCl (LSmeans ± SEM, *n* = 5). ANOVA of NO production (A): *p*_maize_ = 0.673; *p*_treatment_ = 0.825; *p*_injection_ = 0.931; *p*_maize×treatment_ = 0.614; *p*_maize×injection_ = 0.526; *p*_treatment×injection_ = 0.690; *p*_maize×treatment×injection_ = 0.279 and ANOVA of FRA levels (B): *p*_maize_ = 0.329; *p*_treatment_ = 0.207; *p*_injection_ = 0.748; *p*_maize×treatment_ = 0.921; *p*_maize×injection_ = **0.015**; *p*_treatment×injection_ = 0.765; *p*_maize×treatment×injection_ = **0.021**.

Nitric oxide (NO) production, determined as the release of nitrite, is illustrated in Figure 4A. The NO production remained unaffected by all treatments and ranged between 14.3 ± 3.7 µM to 20.0 ± 3.7 µM.

Ferric reducing ability (FRA) is considered as an indicator of the total non-enzymatic antioxidant capacity of serum and is measured as Fe^2+^ equivalents (µM) per L serum. Results of FRA in serum are displayed in Figure 4B. Saline-injected pigs fed diets containing untreated FUS maize showed a higher FRA (FUS−/NaCl: 643.4 ± 86.0 µM) compared to its CON-fed counterparts (CON−/NaCl: 393.0 ± 86.0 µM). In contrast, pigs fed diets containing SoS wet-preserved treated maize exhibited a similar FRA level independent of *Fusarium* toxin and LPS injection, and amounted to 487.1 ± 86.0 µM, 429.4 ± 86.0 µM, 457.6 ± 86.0 µM, and 382.6 ± 86.0 µM in groups CON+/NaCl, FUS+/NaCl, CON+/LPS and FUS+/LPS, respectively. Additionally, the FRA level was increased in untreated CON-fed pigs challenged with LPS (CON−/LPS) with 696.3 ± 86.0 µM and therefore resulted in a three factorial interaction between maize batch, SoS treatment and LPS.

## 3. Discussion

Recently, wet preservation of *Fusarium* contaminated maize with SoS was clearly shown to reduce the DON content of contaminated maize in vitro [4], while the in vivo efficiency of this procedure was tested in a feeding experiment with rearing piglets [12]. While the untreated FUS-diet clearly reduced feed intake and weight gain of piglets, SoS treatment of the FUS-maize could overcome these negative effects resulting in growth performance levels comparable to the control groups [8]. Furthermore, as a specific indicator for the successful detoxification, SoS treatment of *Fusarium* toxin-contaminated maize significantly reduced the exposure to DON as indicated by the reduced toxin residue levels in feed and in various physiological samples of pigs [8,9]. 

Based on the reported unspecific effects of SBS treatment on the liver and the proliferative ex vivo response of porcine PBMC, we hypothesized that SoS treatment of maize would affect biochemical indicators of liver function and integrity, the white blood count, as well the redox status. Therefore, as part of the cited feeding experiment [12] we investigated the interactions between *Fusarium* contaminated maize and SoS treatment in the absence and presence of an LPS- induced acute inflammation in the present study to understand the treatment effects under inflammatory stress conditions.

TNF-α plays an essential role in the acute phase response and represents one of the first pro-inflammatory cytokines which is released in response to LPS, and is therefore suited to verify the intended initiation of a systemic inflammation. Although our data show significantly higher TNF-α concentration in most pigs challenged with LPS, the TNF-α concentration in LPS-challenged pigs fed a CON+ diet (CON+/LPS) did not completely reflect this observation. Looking closer, only one out of five pigs in this group showed a higher TNF-α concentration of 9596 pg/mL, the other four pigs exhibited lower values of TNF-α of approximately 111 pg/mL. This high variation might be due to the timing of blood collection for TNF-α analysis as well as the route of application. The peak TNF-α concentration appears often approximately 1 h after intravenous LPS-infusion [24] in pigs. In the present study blood samples were collected later, 2 h after intraperitoneal LPS-injection. The rationale behind this timing was to consider the time required for LPS-absorption from the abdominal cavity into the blood stream when injecting intraperitoneally rather than intravenously. Taking into account an individual vulnerability as well as LPS-absorption kinetics, it might well be that peak concentrations of TNF-α were not covered in each of the challenged pigs. However, the mean increase in body temperature in group CON+/LPS was comparable to that of the other LPS-challenged groups supporting the view that the systemic inflammation was initiated [25]. Moreover, the cumulative clinical score would rather suggest a less pronounced systemic inflammation in LPS-challenged groups fed SoS treated uncontaminated and contaminated maize. 

Organ weights do not support this interpretation. Viewing data of the emptied stomach weights, it appeared that SoS treatment only of the contaminated maize obviously prevented the LPS-induced weight increase observed in all other groups including group CON+/LPS. While the LPS-associated increase in stomach weight might be due to oedema [26], the mechanism why feeding of SoS treated contaminated maize prevented stomach oedema remains to be clarified but seems to be related to the presence of DONS, both in stomach chyme and in systemic circulation. The LPS-induced increase in spleen weight might be due to a splenic congestion [27] which was not affected by SoS treatment of maize. The lower lung weights detected in groups fed the diets containing the SoS treated contaminated maize probably reflect an effect of chronic feeding while an acute LPS effect was not visible. In addition, neither clinical-chemical nor haematological traits support the view that treatment of maize with SoS influenced the outcome of the LPS-induced systemic inflammation. 

It has been suggested that the alteration of clinical chemistry represents secondary effects of an LPS-induced acute phase reaction [7,24]. In agreement with these reports, in the present study the total bilirubin and γ-GT concentrations were significantly increased in LPS-injected animals indicating cholestasis relative to the saline-injected CON groups. Other indicators of hepatocyte integrity (GLDH, AST) and liver function (albumin concentration) remained unaltered, irrespective of LPS challenge and SoS treatment of maize, leading to the conclusion that the observed LPS-induced effects on the liver were neither modified nor directly influenced by SoS treatment.

The present results demonstrated significantly three factorial interactions for some parameters of white blood counts (the total leukocyte counts, lymphocytes and monocytes). It needs to be stressed however that these observations were characterized by a high individual variation. Feeding of SoS treated FUS maize—which contained mostly DON-sulfonates (DONSs)—caused significant effects and interactions for both segmented and banded neutrophils and would suggest a DONS effect. Previous studies have demonstrated that DONSs are considered less toxic derivatives of DON [4,6,7,8,13,28]. The DONS effect on increased neutrophil counts is not consistent with data from the other part of our study [9], whereby feeding the diets containing SoS treated FUS maize did not show any impact on the neutrophils counts. Such results were also reported for the impact of FUS maize treated with SBS, mono-methylamine and calcium hydroxide in diets for female piglets [29]. In the present experiment LPS injection induced neutropenia in almost LPS-injected piglets with exception in the group FUS+. Looking closer, an increase in neutrophils was found in the group FUS+. Thus, another explanation for the enhancement of neutrophil counts might be due to the increased influx from the bone marrow and/or a decreased apoptosis of neutrophils compared to the control groups. As the DON content in the control groups was very low, this observation is seemingly related to the DONS effect.

Besides the white blood count, the ferric reducing ability (FRA) of serum was influenced by treatment factors in an interactive manner whereby SoS treatment effects appeared to be less consistent. Therefore, both sulfite residues and DONS might have influenced FRA in dependence on SoS treatment and maize batch. It was considered that the antioxidative effect of sulfite is due to the formation of sulfite (SO_3_^2−^) [30]. In animals, sulfite is rapidly reduced by the enzyme sulfite oxidase to sulfate (SO_4_^2−^) [31], which might contribute to this antioxidative capacity of sulfite. On the other hand, sulfite also caused oxidative stress due to sulfite oxidation into a sulfite radical (SO_3_^−■^) [13,30]. Therefore, the effect of sulfite on the redox status in pigs was of interest and examined in the present study. Although our data showed the interaction between FUS maize, SoS treatment and LPS injection for the FRA level, it needs to be stressed that this finding was characterized by high individual variation. Moreover, the NO production, which represents another indicator for oxidative stress, remained unaffected irrespective of SoS treatment and LPS injection leading to the suggestion that SoS treatment of maize did not modulate the redox status, neither in LPS-stimulated nor in unstimulated animals. 

Taken together, we could demonstrate that SoS treatment of maize did not have an impact on our investigated parameters of liver function and integrity, redox status and blood cell counts, with the exception of neutrophil counts that were generally increased in FUS+ fed piglets irrespective of the LPS-induced systemic inflammation. Thus, this effect is most likely due to the presence of DONS in FUS+ diets, but the underlying mechanism for this relative neutrophilia requires further elucidation.

## 4. Materials and Methods

The piglet experiment was performed at the Institute of of Animal Nutrition, Friedrich-Loeffler-Institut, Federal Research Institute for Animal Health, Braunschweig, Germany according to the European Community regulations concerning the protection of experimental animals and was approved by the Lower Saxony State Office for Consumer Protection and Food Safety (file number: 33.92-42502-04-13/1153, date of approval: 11.07.2013).

### 4.1. Experimental Design and Procedures

The present manuscript is part of a comprehensive study, investigating the efficacy of detoxification of DON-contaminated maize with sodium sulfite. Experimental design, SoS treatment in *Fusarium* toxin contaminated maize as well as procedures have been already described in detail in Paulick et al. [8] and Tran et al. [9]. Briefly, a total of eighty male castrated weaned crossbred piglets ((German Landrace × German Large White) × Piétrain) from the Bundeshybridzuchtprogramm (BHZP) with an average initial BW of 7.59 ± 0.92 kg were group-housed (4 pigs/pen) and equally allotted to one of four experimental diets, fed over a period of 42 days: CON− (diet with 10% control maize; 0.09 mg DON/kg and <0.01 mg ZEN/kg feed), CON+ (diet with 10% control maize, wet-preserved with 5 g SoS/kg maize; 0.05 mg DON/kg and <0.01 mg ZEN/kg feed), FUS− (diet with 10% mycotoxin-contaminated maize; 5.36 mg DON/kg; 0.09 mg DONS-2/kg; 0.06 mg DONS-3/kg and 0.29 mg ZEN/kg feed) and FUS+ (diet with 10% contaminated maize, wet-preserved with 5 g SoS/kg maize; 0.83 mg DON/kg; 2.62 mg DONS-2/kg; 1.98 mg DONS-3/kg and 0.27 mg ZEN/kg feed). Diets consisted of 35% barley, 27.3% wheat, 10% maize, 23% soybean meal, 1.5% soya bean oil, 1% mineral and vitamin premix, 2.2% amino acid and phytase. More details about the diets are described in Tran et al. [9]. Feed and water were offered ad libitum.

**Figure 5:**
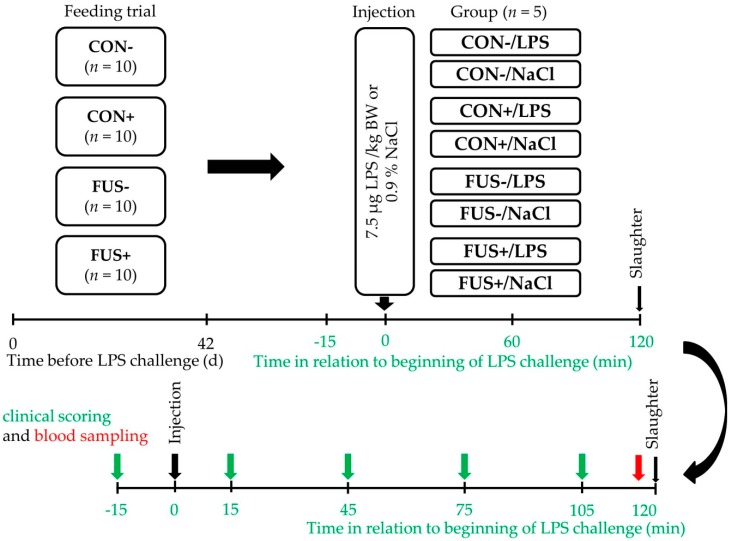
The feeding trial included diets containing 10% control maize (CON−) or *Fusarium* toxin-contaminated maize (FUS−), or sodium sulfite (SoS) wet-preserved treated control (CON+) and FUS maize (FUS+). The feeding trial lasted for 42 days before LPS challenge (upper panel). On day 42, ten pigs of each group were injected intraperitoneally either with 7.5 µg LPS/kg BW (*Escherichia coli* serotype O111:B4, Sigma-Aldrich, Steinheim, Germany, L 2630) or with 0.9% NaCl (volume ~ 6.5 mL/animal). The lower panel displays the LPS challenge, over a period of 2 h, starting 15 min before LPS challenge until 120 min after injection clinical symptoms were recorded and blood samples were taken once direct before slaughter.

At the last day of the feeding experiment, on day 42, ten pigs of each feeding group were used for the LPS challenge. An overview about the LPS challenge is illustrated in Figure 5.

The dose of LPS was chosen for induction of a reliable systemic inflammation as described in previous studies [24,25]. Over a period from −15 min before to 120 min after injection, different clinical signs were scored (Table 4), respiratory rate was taken, and rectal temperature measured using a digital thermometer (Geratherm Rapid GT-195, Geratherm Medical AG, Geschwenda, Germany) at −15, 15, 45, 75 and 105 min. At 120 min, all animals were electrically stunned and blood samples were taken immediately via venipuncture of *Vena cava cranialis*. Then, pigs were sacrificed by exsanguination and organs dissected from the thoracic and abdominal cavity. Weights of emptied stomach, liver (without gallbladder), spleen, kidneys, heart and lungs were recorded.

### 4.2. Analyses

#### 4.2.1. Haematology and Biochemichal Analysis

Blood samples were collected into EDTA-tubes (Sarstedt AG&Co, Nümbrecht, Germany). Red blood cell counts and total leukocytes counts were measured on a Celltac-α (MEK 6450, Nihon Kohden, Qinlab Diagnostik, Weichs, Germany). In order to differentiate white blood cells, blood smears were prepared and stained according to PAPPENHEIM: Slides were fixed and stained in MAY-GRÜNWALD solution for 3 min and subsequently rinsed in distilled water (pH 7.2) to remove staining solution. Then, slides were counterstained in a GIEMSA solution for 15 min and again rinsed in distilled water (pH 7.2). Dried specimens were microscopically analyzed (Nikon Eclipse E200, Nikon Europe b.v., Badhoevedorp, The Netherlands) and at least 200 cells counted per slide (100× magnifications) based on their morphological characteristics. The differential blood counts were firstly calculated as percentage of total leukocytes. Data of differential blood counts are presented in absolute values and were calculated as the total leukocytes multiplied with the proportion of corresponding cell subpopulation.

Further blood samples were collected into serum tubes (Serum Z, Sarstedt AG&Co, Nümbrecht, Germany), left clotting for 60 min at room temperature and 30 min at 30 °C, subsequently centrifuged at 2123× *g* for 15 min (15 °C) and stored at −80 °C until analysis. Serum was analyzed for total protein, albumin, total bilirubin, aspartate-aminotransferase (AST), glutamate-dehydrogenase (GLDH) and γ-glutamyl transferase (γ-GT) using photometric methods (Eurolyser, CCA180, Eurolab, Austria). The cytokine tumor necrosis factor α (TNF-α) was determined by commercially available ELISA (Quantikine^®^ ELISA, R&D systems) using the specific immunoassay kit for pigs.

#### 4.2.2. Oxidative Status

##### Griess Assay

The release of nitric oxide was measured with the Griess assay employing to enzymatic reduction of nitrate to nitrite and reaction of nitrite with Griess reagent. Briefly, 80 µL serums (duplicates) was pipetted in a 96-well MTP, 10 µL nitrate reductase cofactor and 10 µL nitrate reductase enzymes (both Cayman Chemical, Ann Arbor, MI, USA) were added to each well. After three hours incubation in the dark, 100 µL of Griess solution (1% sulfanilamide and 0.1% *N*-(1-naphthyl) ethylenediamine dihydrochloride) were added to each well and incubated for 10 min at room temperature and absorbance was measured at 540 nm (Tecan Infinite^®^ 200, Tecan Group Ltd., Männedorf, Switzerland).

##### Ferric Reducing Ability

The ferric reducing ability (FRA) of serum was measured according to the specifications of Benzie and Strain [32] with in-house modifications. Briefly, 10 µL serums (triplicate) were placed into wells of a 96-well MTP and mixed with 30 µL distilled water (pH 7.2). After incubating for 5 min at 37 °C, 300 µL FRA reagent including ferric (III) chloride hexahydrate, acetate buffer and 2,4,6-tripyridyl-s-triazine (TPTZ) (pre-warmed for 30 min at 37 °C) were added to each well and the absorbance of this mixture was measured at 593 nm for 15 min (Tecan Infinite^®^ 200, Tecan Group Ltd., Männedorf, Switzerland).

### 4.3. Statistics

Generally, data were statistically analyzed with a 2 × 2 × 2-factorial design using PROC MIXED in SAS software (SAS Institute 2013, Cary, NC, USA), presented as least square means (LSMeans) and pooled standard error of means (PSEM). Maize batch (control maize or *Fusarium* toxin-contaminated maize), SoS treatment (with or without SoS) and injection (injection of NaCl or LPS) as well as their interactions were defined as fixed factors. The student’s *t*-test was used for post-hoc testing of differences between LSMeans and considered significant at *p* < 0.05. 

Data of cytokine TNF-α were not normally distributed and thus evaluated using a Mann-Whitney U-test (Statistica 13, Dell Inc., Tulsa, OK, USA). Corresponding data were presented as median and respective ranges (minimum-maximum values).

## Figures and Tables

**Table 1:** Live weight and relative organ weights (g/kg BW) of male castrated piglets fed diets containing control (CON−) or *Fusarium* toxin-contaminated maize (FUS−), or sodium sulfite (SoS) wet-preserved treated control (CON+) and FUS maize (FUS+). Pigs were i.p. injected with either 7.5 µg LPS/kg BW or 0.9% NaCl (LSMeans, *n* = 5).

Maize	Treatment	Injection	BW [kg]	Heart	Liver	Spleen	Kidney	Lung	Stomach
CON	−	NaCl	30.6	4.8	25.5	1.5	4.8	13.0	7.2
CON	−	LPS	29.6	4.7	28.9	2.0	4.6	11.1	8.0
CON	+	NaCl	29.8	4.7	26.3	1.6	4.6	13.8	7.4
CON	+	LPS	30.8	4.7	25.4	1.6	4.3	13.8	8.3
FUS	−	NaCl	25.8	4.8	25.9	1.6	4.8	14.6	9.4
FUS	−	LPS	26.3	4.4	27.4	1.8	4.8	13.8	10.1
FUS	+	NaCl	31.4	4.7	26.1	1.6	4.5	13.1	7.9
FUS	+	LPS	30.4	4.7	26.4	1.7	4.6	10.3	7.9
ANOVA (*p*-value)									
Maize			0.087	0.712	0.897	0.888	0.482	0.995	**<0.001**
Treatment			**0.015**	0.846	0.261	0.141	0.126	0.645	**0.010**
Injection			0.495	0.503	0.181	**0.015**	0.642	0.082	**0.048**
Maize × treatment			**0.023**	0.697	0.524	0.881	0.831	**0.009**	**0.001**
Maize × injection			0.495	0.606	0.834	0.590	0.382	0.580	0.392
Treatment × injection			0.495	0.388	0.081	0.082	0.890	0.973	0.636
Maize × treatment × injection			0.742	0.668	0.319	0.242	0.793	0.211	0.545
PSEM ^#^			1.4	0.2	1.1	0.1	0.2	1.1	0.4

Notes: ^#^ PSEM, Pooled standard error of means, LSMeans, least square means.

**Table 2:** Total and differential white blood cell counts [10^9^/L] of male castrated piglets fed diets containing untreated control (CON−) or *Fusarium* toxin-contaminated maize (FUS−), or sodium sulfite (SoS) wet-preserved treated control (CON+) and FUS maize (FUS+). Pigs were i.p. injected with either 7.5 µg LPS/kg BW or 0.9% NaCl (LSMeans, *n* = 5).

Maize	Treatment	Injection	Leukocytes (10–22) ^Φ^	Lymphocytes (6–16) ^Φ^	Segmented Neutrophils (1–8.2) ^Φ^	Banded Neutrophils (0–1.5) ^Φ^	Monocytes (0–1) ^Φ^	Eosinophils (0–1.3) ^Φ^
CON	−	NaCl	18.7	11.9	4.7	0.5	0.7	0.3
CON	−	LPS	9.8	6.6	2.3	0.4	0.2	0.2
CON	+	NaCl	13.4	8.7	3.7	0.4	0.2	0.3
CON	+	LPS	14.0	10.2	2.6	0.5	0.4	0.3
FUS	−	NaCl	14.4	8.4	4.8	0.6	0.4	0.2
FUS	−	LPS	7.8	6.0	1.3	0.2	0.2	0.1
FUS	+	NaCl	20.8	12.2	6.8	0.9	0.7	0.3
FUS	+	LPS	13.0	8.0	3.7	0.7	0.3	0.2
ANOVA (*p-*value)								
Maize			0.974	0.388	0.091	0.126	0.836	0.218
Treatment			**0.034**	0.062	0.067	**0.028**	0.960	0.258
Injection			**<0.001**	**0.003**	**<0.001**	**0.049**	**0.002**	0.380
Maize × treatment			**0.012**	0.112	**0.011**	**0.019**	**0.022**	0.691
Maize × injection			0.206	0.425	0.095	0.112	0.491	0.843
Treatment × injection			0.092	0.132	0.366	0.299	0.064	0.948
Maize × treatment × injection			**0.033**	**0.012**	0.664	0.809	**0.011**	0.858
PSEM ^#^			1.7	1.1	0.7	0.1	0.1	0.1

Notes: ^Φ^ Reference values according to Kraft and Dürr (2014) [23]; LSMeans, least square means; ^#^ PSEM, Pooled standard error of means.

**Table 3:** Serum biochemical parameters of male castrated piglets fed diets containing untreated control (CON−) or *Fusarium* toxin-contaminated maize (FUS−), or sodium sulfite (SoS) wet-preserved treated control (CON+) and FUS maize (FUS+). Pigs were i.p. injected with either 7.5 µg LPS/kg BW or 0.9% NaCl (LSMeans, *n* = 5).

Maize	Treatment	Injection	Protein [g/L] < 86 ^Φ^	Albumin [g/L] (19–39) ^Φ^	AST ^†^ [U/L] < 35 ^Φ^	γ-GT ^‡^ [U/L] < 45 ^Φ^	Total Bilirubin [µmol/L] < 4.3 ^Φ^
CON	−	NaCl	48.0	32.7	36.8	24.0	3.1
CON	−	LPS	45.5	32.4	46.3	29.1	3.5
CON	+	NaCl	45.0	32.4	39.7	25.4	2.4
CON	+	LPS	45.2	33.0	49.4	24.4	3.0
FUS	−	NaCl	46.0	31.2	49.6	24.0	3.0
FUS	−	LPS	41.1	29.7	50.0	28.1	4.2
FUS	+	NaCl	49.2	34.0	49.0	26.8	2.6
FUS	+	LPS	43.7	32.8	40.8	29.5	3.5
ANOVA (*p-*value)							
Maize			0.583	0.438	0.153	0.259	0.305
Treatment			0.700	0.073	0.744	0.845	0.087
Injection			0.060	0.466	0.334	**0.031**	**0.024**
Maize × treatment			0.181	0.105	0.184	0.130	0.949
Maize × injection			0.220	0.363	**0.028**	0.594	0.345
Treatment × injection			0.755	0.713	0.481	0.130	0.949
Maize × treatment × injection			0.616	0.876	0.464	0.332	0.784
PSEM ^#^			2.3	1.2	4.2	1.7	0.5

Notes: ^Φ^ Reference values according to Kraft and Dürr (2014) [23]; ^†^ AST, Aspartate aminotransferase, ^‡^ γ-GT, γ-glutamyltransferase; LSMeans, least square means; ^#^ PSEM, Pooled standard error of means.

**Table 4:** Clinical score: Cumulative score calculated from all scores of each symptom over the whole observation period (8 symptoms × 4 times, maximum score 88) or of each symptom for every point in time (8 symptoms per time, maximum score 22).

Clinical Symptom	Grade	Score
Respiratory difficulties	None	0
	Low labored breathing	1
	Medium labored breathing	2
	Severe labored breathing	3
	Open-mouth breathing	4
Tremor	None	0
	Low shivering	1
	Medium shivering	2
	Severe shivering	3
	Spasms	4
Conjunctivae	Physiological (pale-rose)	0
	(Rose) red	1
	Red	2
Episcleral vessels	Physiological (not injected)	0
	Slightly injected	1
	Medium injected	2
	Highly injected	3
Cyanosis	None	0
	Low cyanosis	1
	Medium cyanosis	2
	Severe cyanosis	3
Vomiting, retching	None	0
	Smacking, foam-forming, retching	1
	Vomiting of slime	2
	Vomiting of feed/digesta	3
	Vomiting of slime and feed/digesta	4
Dermographism	None	0
	Skin colouring pattern present	1
Teeth gnashing	None	0
	Teeth gnashing present	1
Maximum clinical score (each animal each time point)		22
Maximum clinical score (each animal for all time points)		88

## Supplementary Materials

The following are available online at http://www.mdpi.com/2072-6651/10/10/419/s1, Table S1: Red haemogram of male castrated piglets fed diets containing untreated control (CON−) or Fusarium toxin contaminated maize (FUS−), or sodium sulfite (SoS) wet-preserved treated control (CON+) and FUS maize (FUS+). Pigs were i.p. injected with either 7.5 µg LPS/kg BW or 0.9% NaCl (LSMeans, *n* = 5).
