# Supplementary material for: Effects of a Fusarium Toxin-Contaminated Maize Treated with Sodium Sulfite on Male Piglets in the Presence of an LPS-Induced Acute Inflammation
Source: Toxins (Basel). 2018 Oct 18;10(10):419. doi: 10.3390/toxins10100419 (PMC6215154; doi:10.3390/toxins10100419)
Supplement: Supplementary file 1 [file toxins-10-00419-s001.pdf]

Supplementary Materials: Effects of a *Fusarium* Toxin-Contaminated Maize Treated with Sodium Sulfite on Male Piglets in the Presence of an LPS-Induced Acute Inflammatio

Anh-Tuan Tran, Jeannette Kluess, Andreas Berk, Marleen Paulick, Jana Frahm, Dian Schatzmayr, Susanne Kersten and Sven Dänicke

Supplementary Table S1. Red haemogram of male castrated piglets fed diets containing untreated control (CON−) or *Fusarium* toxin contaminated maize (FUS−), or sodium sulfite (SoS) wet-preserved treated control (CON+) and FUS maize (FUS+). Pigs were i.p. injected with either 7.5 µg LPS/kg BW or 0.9% NaCl (LSMeans, n = 5).

Maize	Treatment	Injection	RBC* [10^6/µL] (5.8-8.1)$^{\Phi}$	HGB** [g/dL] (10.8–14.8)$^{\Phi}$	HCT† [%] (33–45)$^{\Phi}$	MCV§ [fL] (50–65)$^{\Phi}$	MCH$ [pg] (17–21)$^{\Phi}$	MCHC‡ [g/dL] (30–35)$^{\Phi}$	RDW# [%] (0–50)$^{\Phi}$
CON	−	NaCl	6.5	12.4	35.7	54.6	19.1	35.0	17.4
CON	−	LPS	6.8	13.4	37.9	55.6	19.6	35.3	17.0
CON	+	NaCl	6.2	11.7	34.4	55.9	19.2	34.3	17.5
CON	+	LPS	6.4	13.7	36.4	57.3	21.7	34.6	16.3
FUS	−	NaCl	6.0	11.6	34.4	57.7	19.3	33.6	18.4
FUS	−	LPS	6.4	12.2	35.8	55.7	18.9	33.9	17.2
FUS	+	NaCl	6.3	12.4	35.3	56.2	19.8	35.3	17.4
FUS	+	LPS	6.5	12.4	36.0	55.0	19.0	34.4	17.0
ANOVA (*p*-value)									
Maize			0.305	0.087	0.396	0.691	0.320	0.468	0.309
Treatment			0.541	0.664	0.616	0.796	0.263	0.753	0.287
Injection			0.071	**0.027**	0.072	0.776	0.499	0.966	0.059
Maize × treatment			0.054	0.290	0.256	0.120	0.519	0.204	0.727
Maize × injection			0.764	0.130	0.536	0.087	0.088	0.700	0.933
Treatment × injection			0.616	0.823	0.770	0.681	0.519	0.648	0.952
Maize × treatment × injection			0.826	0.302	0.888	0.894	0.336	0.690	0.309
PSEM°			0.2	0.5	1.2	1.1	0.9	1.0	0.6

Notes: $^{\Phi}$ Reference values according to Kraft and Dürr (2014); * RBC, Red blood cells, ** HGB, Haemoglobin, † HCT, Haematocrit, § MCV, Mean cell volume, $ MCH, Mean corpuscular haemoglobin, ‡ MCHC, Mean corpuscular haemoglobin concentration, # RDW, Red cell distribution width; LSMeans, least square means; ° PSEM, Pooled standard error of means.
